# *Chryseobacterium indologenes* sepsis in a pediatric patient

**DOI:** 10.31744/einstein_journal/2025RC1200

**Published:** 2025-04-07

**Authors:** Felipe Krakauer, Raquel Pissiguelli, Marina Moranduzzo, Bruna Marques Tofaneto, Sophia Bastos Correa da Costa, Maryleen Brigitte Muñoz Guzman, Gabriel Frizzo Ramos, Saulo Brasil do Couto, Rafael Yanes Rodrigues da Silva, Luís Felipe Batista Hiar, Luisa Zagne Braz

**Affiliations:** 1 Hospital Israelita Albert Einstein Faculdade Israelita de Ciências da Saúde Albert Einstein São Paulo SP Brazil Faculdade Israelita de Ciências da Saúde Albert Einstein, Hospital Israelita Albert Einstein, São Paulo, SP, Brazil.; 2 Hospital Israelita Albert Einstein Hospital Municipal da Vila Santa Catarina Dr. Gilson de Cássia Marques de Carvalho São Paulo SP Brazil Hospital Municipal da Vila Santa Catarina Dr. Gilson de Cássia Marques de Carvalho; Hospital Israelita Albert Einstein, São Paulo, SP, Brazil.

**Keywords:** Chryseobacterium, Flavobacteriaceae, Drug resistance, multiple, Bacteremia, Cross infection, Breast-milk substitutes, Child

## Abstract

*Chryseobacterium indologenes* is a Gram-negative aerobic bacillus commonly found in nosocomial environments, particularly in patients with prolonged hospital stays or those requiring long-term invasive devices. It primarily affects elderly and immunocompromised individuals. This microorganism is associated with multidrug resistance, which is a crucial factor in treatment decisions. Here, we report a case of *C. indologenes* infection in an infant following the ingestion of milk formula diluted with untreated water.

## INTRODUCTION

*Chryseobacterium indologenes* is a Gram-negative, non-fermenting, oxidase- and catalase-positive^(1)^ aerobic bacillus, first described by Vandamme et al in 1994.^(2)^ It is commonly found in soil and water.^(3)^ This bacterium exhibits natural resistance to several antibiotics,^(1)^ which can complicate treatment and necessitate the use of more potent drugs, potentially leading to adverse effects.

Although human infections are rare, *C. indologenes* possesses significant infectivity due to its ability to form a biofilm, particularly in areas exposed to water, such as sinks, catheters, and probes. This characteristic facilitates its spread as a nosocomial pathogen.^(4)^ The primary types of infections include hematogenous infections, respiratory tract infections, central nervous system infections, intra-abdominal infections, skin infections, and device-associated infections.^(1)^ The most vulnerable populations include elderly individuals with comorbidities, immunocompromised patients, those with prolonged hospitalization, and individuals receiving prolonged broad-spectrum antibiotic therapy.^(1,4)^

Reports of *C. indologenes* infections in the pediatric population are rare, with most documented cases involving nosocomial transmission through mechanical ventilation or other invasive procedures.^(5,6)^ The disease course is generally benign, with full resolution and no documented recurrence when appropriate antibiotic therapy, guided by susceptibility testing, is administered.

Here, we present a case of *C. indologenes* infection in an infant treated at a general hospital in São Paulo. The patient had no prior hospitalizations and presented with acute diarrhea for 10 days. A multidrug-resistant strain of *C. indologenes* was isolated from blood cultures, highlighting the significance of community-acquired infection in this case.

## CASE REPORT

A 7-weeks-old male nursling, a full-term monozygotic twin with no complications during pregnancy or birth, was brought to the emergency department by his parents due to severe diarrhea lasting 10 days, accompanied by a single episode of vomiting on the last day and reduced food intake. The parents reported an approximate weight loss of one kilogram since symptom onset, with no fever. His twin brother remained asymptomatic. The patient had been fed with milk formula diluted with untreated tap water and undiluted whole cow's milk since birth.

Upon arrival, the infant was in a regular general condition for his gestational age but presented normotensive, tachycardic, hypothermic, and moderate respiratory distress. Physical examination revealed severe dehydration, with cold, slightly sticky extremities; a depressed anterior fontanel; sunken eyes; xerostomia; and bilaterally dry, dull sclerae.

Given the critical condition, the patient was admitted to the pediatric intensive care unit (ICU). Fluid resuscitation was initiated with a total volume expansion of 45 mL/kg (160 mL) of 0.9% saline, administered in two stages, with a good clinical response. Laboratory tests revealed metabolic acidosis, which arterial blood gas analysis with pH values = 6.97, pCO_2_ 13 mmHg, pO_2_ 99 mmHg, BE −26.8 / HCO_3_ 3.1 mEq/L, O_2_ saturation 93%. Additionally, laboratory findings suggested acute kidney injury, with creatinine values of 0.98 mg/dL, urea of 136 mg/dL, and potassium of 6.2 mEq/L. The complete blood count (CBC) showed: Hemoglobin: 8.5 g/dL; hematocrit: 25.2%; Leukocytes 14370/mm^3^ with 55.5% neutrophils, 0.5% eosinophils, 32% lymphocytes, and 12% monocytes; platelets: 616000/mm^3^. Inflammatory markers revealed a C-reactive protein (CRP) level of 3.6 mg/dL. Stool tests for adenovirus and rotavirus were negative.

Empirical antibiotic therapy with ceftriaxone (100mg/kg/day) was initiated, resulting in a good clinical response. The patient remained in the ICU for 5 days, showing both clinical and laboratory improvement, and was subsequently transferred to the pediatric ward.

During hospitalization, three blood cultures and one stool culture were performed. The first blood culture, collected at admission, tested positive for a Gram-negative bacillus after 22 hours of incubation, with C. indologenes isolated two days later. The final identification was confirmed within one additional day. The antibiotics susceptibility profile showed sensitivity to Cefepime (1:4 dilution), resistance to meropenem (1:32 dilution), and "not applicable" for sulfamethoxazole-trimethoprim (0.25 dilution). The second blood culture obtained 4 hours after the first, was collected after empirical antibiotic therapy had already been initiated, alongside the stool culture. Both tests yielded negative results. The third blood culture, performed 5 days after admission as a control, also tested negative.

Due to the resistance profile observed in the antibiogram, ceftriaxone was replaced with cefepime (150mg/kg/day). Following the antibiotic switch, laboratory tests indicated improvement in metabolic acidosis, with arterial pH: 7.56, pCO_2_: 27mmHg, pO_2_ 77mmHg, BE: 1.9; HCO_3_: 24.2mEq/L and hemoglobin saturation of 97%; of acute kidney injury with; creatinine 0.26mg/dL, potassium: 5.2mEq/L and urea: 12mg/dL; and infectious parameters with procalcitonin: 0.13 ng/mL, C-reactive protein: 1mg/L, Arterial lactic acid: 18mg/dL, hemoglobin: 7.8g/dL, hematocrit 22.7%, leukocytes: 13540/mm3 with 42.5% neutrophils, 2.7% eosinophils, 46.8% lymphocytes and 7.9% basophils and platelets: 464000/mm^3^. The patient demonstrated weight gain and improved food intake, leading to hospital discharge after completing 10 days of antibiotic therapy.

This study was approved by the Research Ethics Committee of *Hospital Israelita Albert Einstein* (CAAE: 78336624.0.0000.0071; # 6.756.812).

## DISCUSSION

Nosocomial infections caused by C. *indologenes* are more prevalent among patients requiring external devices, particularly long-term catheters and ventilatory support.^(3-5)^ In this case, ingestion of untreated water used to prepare infant formula, combined with the patient's young age, likely contributed to contamination with C. *indologenes,* leading to severe metabolic acidosis and acute kidney injury.

This case is among the few reported in the literature where *C. indologenes* was isolated from a positive blood culture in a patient without prior hospital exposure or long-term device use, suggesting a probable extra-hospital source of infection. This hypothesis is supported by the early blood culture positivity, as bacterial growth was detected within just 22 hours of hospital admission. However, it is important to consider the possibility of laboratory culture contamination, despite the absence of prior records of *C. indologenes* in the hospital's microbiological surveillance and the patient's clinical course, which did not suggest contamination.

The intrinsic multidrug resistance of *C. indologenes* is a key factor in treatment selection. According to the literature, the most effective antimicrobials are quinolones (gatifloxacin and levofloxacin) and sulfamethoxazole-trimethoprim.^(7)^ However, in this case, the pathogen demonstrated resistance to sulfamethoxazole-trimethoprim, and the use of certain antibiotics in pediatric patients is limited, further restricting therapeutic options. Notably, unlike previously described cases, this patient showed mild clinical improvements with ceftriaxone, suggesting a potential in *vivo* response not observed *in vitro*. Additionally, most reported cases of *C. indologen*es infection occur in elderly, critically ill patients in intensive care settings, often involving polymicrobial infections, which can further complicate targeted antimicrobial therapy.^(3)^

Despite the increasing number of reported cases, there are no standardized treatment protocols for *C. indologen*es infection, potentially delaying the initiation of appropriate therapy. Therefore, individualized treatment decisions based on antibiotic susceptibility testing are essential.

Given that *C. indologen*es is naturally found in soil and water, effective preventive measures should focus on maintaining personal hygiene and ensuring the consumption of treated drinking water to reduce the risk of contamination and severe infection, particularly considering the pathogen's multidrug resistance profile.

## CONCLUSION

Although *C. indologenes* infections are rarely reported in the literature, particularly in pediatric patients, they can be potentially fatal due to the pathogen's intrinsic multidrug resistance. Therefore, prophylaxis, early identification, and prompt treatment are crucial in managing these infections. With the increasing number of reported cases, establishing a standardized treatment protocol could help provide unified and effective management strategies. However, further research is needed to better characterize the pathogen's behavior, explore additional contamination routes, and identify potential adjuvant therapies that could enhance treatment outcomes.
